# ID22 – the high-resolution powder-diffraction beamline at ESRF

**DOI:** 10.1107/S1600577523004915

**Published:** 2023-07-18

**Authors:** Andrew Fitch, Catherine Dejoie, Ezio Covacci, Giorgia Confalonieri, Ola Grendal, Laurent Claustre, Perceval Guillou, Jérôme Kieffer, Wout de Nolf, Sébastien Petitdemange, Marie Ruat, Yves Watier

**Affiliations:** a ESRF, 71 Avenue des Martyrs, CS40220, 38043 Grenoble Cedex 9, France; Advanced Photon Source, USA

**Keywords:** powder X-ray diffraction, high resolution, ESRF, PDF analysis

## Abstract

Following Phase 2 of the upgrade of the ESRF in which the storage ring was replaced by a new low-emittance ring along with many other facility upgrades, the status of ID22, the high-resolution powder-diffraction beamline, is described. The beamline has an in-vacuum undulator as source providing X-rays in the range 6–75 keV. ID22’s principle characteristics include very high angular resolution as a result of the highly collimated and monochromatic beam, coupled with a 13-channel Si 111 multi-analyser stage between the sample and a Dectris Eiger2 X 2M-W CdTe pixel detector. The detector’s axial resolution allows recorded 2θ values to be automatically corrected for the effects of axial divergence, resulting in narrower and more-symmetric peaks compared with the previous fixed-axial-slit arrangement. The axial acceptance can also be increased with increasing diffraction angle, thus simultaneously improving the statistical quality of high-angle data. A complementary Perkin Elmer XRD1611 medical-imaging detector is available for faster, lower-resolution data, often used at photon energies of 60–70 keV for pair-distribution function analysis, although this is also possible in high-resolution mode by scanning up to 120° 2θ at 35 keV. There are various sample environments, allowing sample temperatures from 4 K to 1600°C, a capillary cell for non-corrosive gas atmospheres in the range 0–100 bar, and a sample-changing robot that can accommodate 75 capillary samples compatible with the temperature range 80 K to 950°C.

## Abstract 2

The status of the high-resolution powder-diffraction beamline ID22 at ESRF is described.

## 1. Introduction

The ESRF has operated a dedicated high-resolution powder-diffraction beamline for users since May 1996. The beamline is characterized by having high angular resolution, resulting from the combination of the high collimation of the X-ray beam emitted from the storage ring, wavelength selection via a cryogenically cooled Si 111 channel-cut monochromator crystal, and the use of a 13-channel Si 111 multi-analyser stage between the sample and the detector. To reach the detector, diffracted photons must satisfy the stringent Bragg condition imposed by an analyser crystal, whose Darwin width is, for example, ∼4 × 10^−4^ degrees at 35 keV. As well as defining the angular acceptance of diffracted X-rays, the analyser crystals also make peak positions insensitive to a number of displacement-type aberrations that affect standard Debye–Scherrer or Bragg–Brentano arrangements (Cox *et al.*, 1983 ▸, 1986 ▸; Hastings *et al.*, 1984 ▸; Cox, 1992 ▸). This is because an analyser crystal defines a true angle of diffraction rather than inferring that angle via the position of a slit or pixel in a position-sensitive detector. An analyser crystal also restricts the volume around the sample from which photons can be transmitted to the detector, thus reducing scattering from air or sample environment, and, by acting as a photon-energy filter, also suppresses background from sample fluorescence.

The beamline was originally built as bending-magnet beamline BM16 (Fitch, 1995 ▸, 1996 ▸) and operated with monochromatic X-rays with available energies in the range 5–40 keV, the high limit imposed by the cut-off of the vertically collimating Rh-coated mirror before the monochromator. Experiments were typically conducted with a beam size of 5 mm × 1 mm [horizontal (h) × vertical (v)] at the sample and a wavelength of ∼0.8 Å (15.5 keV). To improve efficiency in detecting the diffracted photons, the first multi-analyser stage, comprising nine Ge 111 analyser crystals spaced with two-degrees intervals, was conceived (Hodeau *et al.*, 1998 ▸). In June 2002 the beamline was upgraded and relocated to Sector ID31 (Fitch, 2004 ▸), benefitting from the increased flux, collimation and energy range of 6–62 keV provided by three undulators, each 1.6 m long with a minimum magnetic gap of 11 mm. Two of these had a magnetic period of 32 mm (u32), and the third had a period of 35 mm (u35). The marked increase in flux resulted in a decrease in scanning times, from ∼8 h to a few tens of minutes to record a high-resolution powder-diffraction pattern, with a beam of 2 mm × 1 mm (h × v) at 31 keV (∼0.4 Å). The ability to work routinely at energies above 30 keV offered the possibility to study capillary samples containing X-ray absorbing heavy elements and to collect high-angle data of adequate quality in a few hours for pair-distribution function (PDF) analysis. In early 2014, during Phase 1 of the ESRF’s upgrade program, the beamline was transferred to Sector ID22, with an increase in the operating range to 75 keV provided by a 2 m-long in-vacuum undulator (u23) with a minimum gap of 6 mm, supplemented by an ex-vacuum u35. With ID22 being located on a high-β section of the former ESRF ring, a highly collimated beam of size 1 mm × 1 mm at 31 keV was delivered to the sample, improving further the high-resolution capabilities of the beamline. A complementary Perkin Elmer XRD1611 medical-imaging detector (41 cm × 41 cm) was added for collecting data with higher statistical quality but lower angular accuracy and resolution, particularly at energies above 60 keV for PDF analysis (Dejoie, Autran *et al.*, 2018 ▸), or for texture analysis.

Phase 2 of the ESRF’s upgrade program involved the replacement of the original storage ring during 2019 with an entirely new one, the ‘extremely bright source’, EBS (Raimondi, 2016 ▸; Raimondi *et al.*, 2023 ▸), designed to have a smaller horizontal emittance, reduced from 4 nm to about 134 pm, thus leading to a significant increase in brightness. For ID22, as a powder-diffraction beamline with relatively simple optics and using a large beam incident on the sample (typically 1 mm × 1 mm), the EBS upgrade has resulted in an increase in flux at the sample of about a factor two, and a reduction of the monochromatic-beam size to 0.6 mm × 0.8 mm (FWHM, h × v) at 31 keV.

Following the restart of user operation in August 2020, we report on the beamline’s current status and discuss some of the possibilities for performing powder-diffraction experiments. A single in-vacuum u26 undulator has been installed as ID22’s source, and the diffractometer equipped with a new detector arm with a Dectris Eiger2 X 2M-W CdTe pixel detector (Donath *et al.*, 2023 ▸) intercepting the X-rays transmitted by the 13 crystals of a new multi-analyser stage, replacing the previous nine-channel version and corresponding array of scintillation counters. *BLISS* (Guijarro *et al.*, 2018 ▸), the in-house-developed ESRF software for beamline control and data acquisition, has replaced *SPEC*, used prior to the EBS upgrade. Routine high-resolution data are generally measured at photon energies above 30 keV, allowing spinning capillaries to be used for all powder samples including those containing X-ray absorbing heavy elements. Spinning capillaries help reduce effects due to preferred orientation, so peak intensities, as well as their positions, are accurate, unless other sample features are present, such as significant graininess. The beamline has a range of sample environments, allowing routine studies in the range 4 K to 1600°C, or under gas pressures of 0–100 bar, and a robotic sample changer which allows a series of 75 samples to be run automatically.

## 2. Configuration

### 2.1. Optics

**Figure 1:**
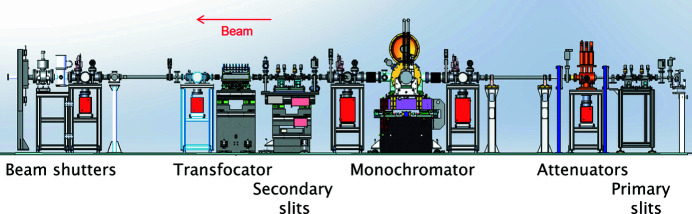
Arrangement of the ID22 beamline optics.

As its source the beamline has a 2.5 m-long in-vacuum u26 undulator, transferred from the ID16B beamline (Martínez-Criado *et al.*, 2016 ▸), giving an energy range of 6–75 keV (λ ≃ 2.1–0.165 Å). The principal optical components, Fig. 1 ▸, are a monochromator, and a transfocator for focusing the monochromatic beam if desired. The monochromator consists of a cryogenically cooled channel-cut Si 111 crystal with a 4 mm channel, which covers the beamline’s entire energy range. The transfocator has 185 paraboloidal aluminium lenses, 200 µm radius of curvature, which can focus the beam onto the medical-imaging area detector to improve data quality, or at the sample position if spatial resolution down to 50 µm × 50 µm is required, *e.g.* in experiments mapping residual strain in a sample. For high-resolution powder-diffraction studies, which make up the majority of experiments, the unfocused monochromatic beam passes directly to the sample, which has a typical intensity of ∼1.1 × 10^13^ photons mm^−2^ s^−1^ at 35 keV (wavelength 0.354 Å, one that is routinely employed) and 200 mA ring current.

### 2.2. Experiments hutch

**Figure 2:**
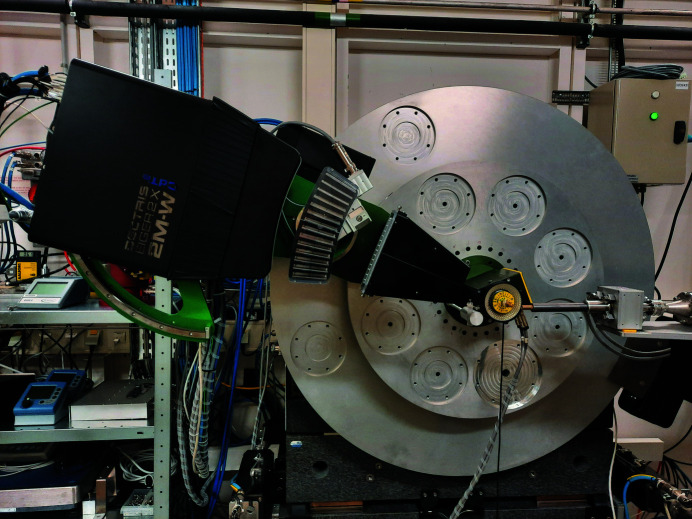
The high-resolution powder diffractometer, equipped with its fast spinner, 13-channel Si 111 multi-analyser stage and Dectris Eiger2 X 2M-W CdTe pixel detector.

There are two instruments for measuring powder-diffraction patterns in the experiments hutch: the high-resolution powder diffractometer, Fig. 2 ▸, and, downstream of this, a large medical-imaging detector. The diffractometer is of a heavy-duty, high-accuracy construction and was supplied by LAB Motion Systems, Leuven, Belgium. This diffractometer replaced the original machine, manufactured by RPI (Bath, UK), after more than 20 years’ service at BM16, ID31 and ID22 before 2017. There are two concentric horizontal axes, with a nominal angular precision of 5 × 10^−6^ degrees provided by the on-axis encoder for the detector-bearing 2θ circle, which is driven by a servo motor via a LAB motion control system, slave to an ESRF Icepap (Janvier *et al.*, 2014 ▸). The ω circle is driven by a stepper motor via an Icepap, with a nominal on-axis encoder precision of 10^−5^ degrees. Air bearings operating at a pressure of 5 bar ensure the smooth rotation of these axes. The diffractometer carries a multi-analyser stage comprising 13 Si 111 analyser crystals each separated from its neighbour by ∼2°, an increase on the nine crystals used on BM16 (Ge 111), ID31 (Si 111) and ID22 (Si 111) until July 2021. The X-rays that are transmitted by the analyser crystals are recorded by a Dectris Eiger2 X 2M-W CdTe pixel detector (Donath *et al.*, 2023 ▸) via 13 zones of interest (Dejoie, Coduri *et al.*, 2018 ▸). The Eiger detector has replaced the bank of nine fast Cyberstar scintillation counters (LaBr_3_ scintillators) used previously. The detector arm can be scanned over the range −40–140° 2θ, corresponding to the position of the middle multi-analyser crystal, and hence a full data range of −52–152° once the angular offsets to the outer crystals are considered (±12°).

The Eiger detector’s active area is 38.4 mm × 311.1 mm (512 × 4148 pixels of size 75 µm × 75 µm) and can optionally be used without the multi-analyser stage as a stand-alone detector, increasing the data acquisition rate at the expense of the angular resolution and accuracy afforded by the analyser crystals. In this mode, the detector covers a range of ∼25° but can be scanned or positioned anywhere within the nominal range of the diffractometer.

The medical-imaging detector is a Perkin Elmer XRD1611, based on an amorphous Si substrate and CsI:Tl scintillator, with 4096 × 4096 pixels of size 100 µm × 100 µm. It is used mainly at higher photon energies for PDF analysis. Its distance from the sample is adjustable in the range 0.38–2 m, allowing a diffraction pattern to be recorded up to 50° 2θ, corresponding to *Q* ≃ 30 Å^−1^ at an energy of 70 keV. The detector is also used for the measurement of texture, *e.g.* in clay samples (Kühn *et al.*, 2021 ▸).

For high-resolution and most area-detector experiments, samples held in thin-walled glass capillaries are mounted on a spinner on the axis of the powder diffractometer. Three spinning devices are available: (i) a routine high-speed spinner allowing spinning speeds of up to 1000 rev min^−1^ for standard samples; (ii) a slower spinner fitted with a goniometer head for mounting more awkward samples where adjustment of the orientation is required – this is the spinner used for mounting the beamline’s gas capillary cell, and can be oscillated as spinning is not possible with a connected gas supply; (iii) a dual-axis Gandolfi spinner, allowing spinning speeds of 6000 rev min^−1^ about an inclined axis, which is mounted on a slower stage (31 rev min^−1^) coaxial with the diffractometer axis. This device is useful for studies of grainy samples to give a better powder average. All three spinners allow translation of the capillary along its axis, by up to 45 mm in the case of the routine high-speed spinner, permitting significant flexibility in the length and filling of the capillaries, and allowing diffraction patterns to be collected from up to 40 positions along the capillary when dealing with radiation-sensitive samples, such as many organic or organometallic materials. In such a case, the diffraction pattern is scanned quickly at a speed of up to 30° 2θ per minute, then the capillary is translated by ∼1 mm to expose fresh sample to the beam, and the cycle repeated (Cockcroft & Fitch, 2008 ▸).

### 2.3. Sample environments

Powder diffraction frequently involves the investigation of samples evolving with temperature, time, atmosphere, pressure *etc*. Routine sample environments at ID22 include an Oxford Cryosystems Cryostream 7+ nitro­gen blower (80–500 K), a hot-air blower (up to ∼950°C), a liquid-helium flow cryostat (van der Linden *et al.*, 2016 ▸) for spinning capillary samples down to 4 K, a mirror furnace with three 150 W halogen bulbs focused on the sample for temperatures up to 1600°C, now complemented by an induction furnace developed at the sample environment laboratory of the ESRF, a gas cell (Brunelli & Fitch, 2003 ▸), controlled by a Druck PACE controller for non-corrosive gases up to 100 bar (replacing an earlier system; Hill, 2013 ▸), and a manual rig for mildly corrosive gases up to 10 bar. Measurements under reduced pressure down to a few mbar are also possible with a turbo pump connected to the system. Experiments involving gas sorption or desorption can be combined with variable temperature via the Cryostream or hot-air blower.

The ‘DynaFlow’ cryostat (van der Linden *et al.*, 2016 ▸) is regulated by a Lakeshore 340 controller, in combination with two downstream Aahlborg GFC mass flow controllers in parallel (2 and 50 l min^−1^, respectively) that vary the helium flow depending on the set temperature via a locally written algorithm. Once the temperature is close to the set point, the helium flow is gradually adjusted so that the temperature controller’s power is in the range 20–40% of full output. This ensures that helium consumption is minimized while keeping sufficient cooling power to allow stable operation. When aiming for temperatures below 50 K, it is necessary either to seal the capillary under He gas or to leave the capillary unsealed to allow He exchange gas to enter the capillary and help remove the energy absorbed from the beam by the sample (Fitch, 2019 ▸). Sealing the capillary under air, argon, nitro­gen, *etc*. leads to condensation of the gas in the capillary and inefficient heat removal with unpredictable effects on the temperature of the sample under the beam, resulting in spurious peak shifts and peak broadening. It is sometimes necessary in addition to attenuate the incident beam to manage beam-heating effects. For the hot-air blower, the air flow is reduced automatically by a GFC controller at temperatures above 900°C to ensure that these temperatures can be attained by the sample. The air flow can also be automatically increased to accelerate cooling down.

There are translation and rotation tables for positioning samples in the beam, *e.g.* for residual strain mapping. A compact Admet strain rig can also be mounted, operating at up to 5 kN in tension or compression. The open access to the diffractometer and the accompanying motorized tables allow complicated experimental setups to be mounted by visiting user groups as necessary, *e.g.* Metcalfe *et al.* (2019 ▸). Generally, sample environments are integrated with the beamline control system, allowing series of measurements to be programmed, executed and monitored without further manual intervention.

The robotic sample changer can handle 75 capillary samples, and is compatible with both the high-resolution and medical-imaging detector configurations using the standard fast spinner, as well as the Cryostream and hot-air blower as sample environments. Thus comprehensive series of measurements can be defined, with a number of samples measured over different temperature ranges, as required.

## 3. Data acquisition and processing

### 3.1. High-resolution mode

#### 3.1.1. General approach

When measuring a high-resolution powder-diffraction pattern, the detector arm is scanned continuously at a speed usually in the range 0.5–30° min^−1^ depending on the nature of the experiment and the sample, and the detector is read by default every (30/scan-speed) ms, corresponding to an angular increment of 0.0005°. Faster or slower read frequencies can be set; for example, for measuring powder-diffraction standards such as NIST Si 640c or LaB_6_ 660c, which have particularly narrow peaks, the read frequency may be doubled or trebled to ensure sufficient sampling of the peak profile. The maximum readout frequency is 1 kHz, with negligible dead-time. The scan range of the central channel is chosen so that the most important parts of the pattern are covered by all 13 channels of the detector, *e.g.* by scanning from −10° to 45°, all channels pass through the range 2–33° 2θ, and the highest-angle detector will record data up to 57°. For the quickest measurements, the detector can be scanned by only 2.2° to cover 26° 2θ, with a different detector contributing to each ∼2° segment, and allowing a small degree of overlap to avoid any gaps in the pattern. The offsets between detector channels are in the range 1.84–2.12°, the imperfect spacing stemming from the way the crystals are glued onto their support with high-temperature vacuum grease to avoid inducing strain. Perfect angular spacing, despite its appeal, is not essential and, in any case, the accurate spacing must be determined with high precision using a standard sample to ensure that the narrowest peak widths are maintained when data from different channels are combined (see below).

The X-rays transmitted by the analyser crystals arrive on specific regions of the Eiger detector (Dejoie, Coduri *et al.*, 2018 ▸). The detector is operated in difference mode, in which two energy thresholds are defined: the first at half the value of the energy of the monochromatic beam, and the second at 1 keV above. To be counted, a photon’s energy must exceed the lower threshold, thus filtering out low-energy electronic noise. In difference mode, counts with energies above the higher threshold are subtracted from those above the lower threshold, thus eliminating any higher harmonics transmitted by the monochromator and analyser crystals (λ/3, λ/4, *etc*.), and cosmic rays.

#### 3.1.2. Basic configuration

In the simplest mode of acquisition, 13 regions of interest of size 4 mm × 2 mm (h × v) are defined straddling the centreline of the detector and the photons arriving in these regions are recorded. The arrangement simulates having 13 standard detectors (such as scintillation counters) with 4 mm × 2 mm entrance slits to suppress parasitic scattering arriving by other routes. Following data collection from a sample, which often includes more than one scan over the same or different angular ranges, the counts recorded from the 13 channels and from the different scans are combined to produce the equivalent step scan in steps (bins) of a size that is appropriate for the crystallinity of the sample. Data points from different channels are summed into the relevant output bins, or partitioned over more than one bin if crossing the boundary between neighbouring bins, via the local software *id22sum* (Wright *et al.*, 2003 ▸) which has been used in various forms since the days of BM16. Highly crystalline samples may need a step of 0.001° (or less for the NIST standards), whereas less crystalline materials will be combined more coarsely, steps of 0.002–0.005°, as appropriate. Signals from the 13 channels must be combined taking into account their exact angular offsets, their relative efficiencies, and the evolution of the incident beam intensity during the measurement, followed via a beam monitor. The offsets and efficiencies are determined from a Si or LaB_6_ standard, by ensuring that the signals measured by all 13 channels superimpose as closely as possible.

#### 3.1.3. Exploiting axial resolution

**Figure 3:**
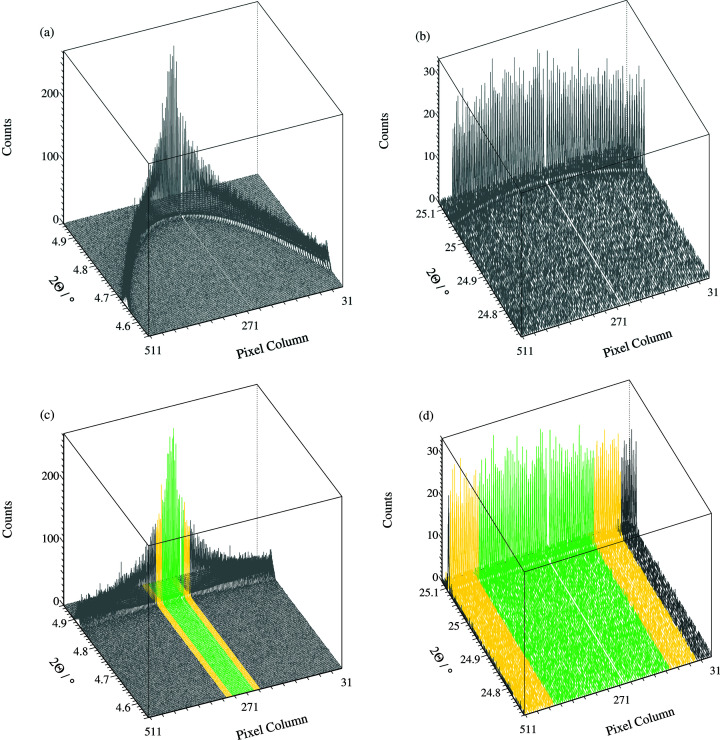
(*a*) LaB_6_ 100 and (*b*) 510/431 peaks as collected illustrating the decrease in apparent diffraction angle with axial divergence; (*c*, *d*) the same, after correction, estimated intrinsic broadening (due to the curvature of the Debye–Scherrer cone and the finite sizes of the beam and receiving pixel) < 0.002°(green) and < 0.003° (yellow). By correcting the angular scale and filtering on intrinsic broadening, higher overall angular resolution is obtained with improved statistics at higher angles.

A newly developed mode of acquisition makes use of the spatial resolution of the Eiger detector to record the axial position of each photon transmitted by an analyser crystal arriving on the detector. For each of the 13 channels, 512 regions of interest across the detector are defined of size 0.075 mm × 1.5 mm (1 × 20 pixels, h × v). The greater the axial deviation of a diffracted photon from the direction of the incident beam, the lower the angle of the detector arm at which the Bragg condition at the analyser crystal is satisfied, causing the well known asymmetry of peak shapes at low diffraction angles. As described by Fitch & Dejoie (2021 ▸) (see supporting information for a minor update), the axial distance from the vertical plane of diffraction can be used to correct the apparent diffraction angle, given by the angle of the detector arm, to the true 2θ angle of diffraction by the sample into the Debye–Scherrer cone, thus reducing the low-angle peak asymmetry and reducing the peak widths overall. Owing to the curvature of the Debye–Scherrer cone, the size of the incident beam and the receiving pixel of the detector, there is in addition an intrinsic broadening to the peaks which increases with axial distance. This broadening can be estimated and used to filter which data with corrected 2θ values are included in the summed diffraction pattern. At higher diffraction angles, the curvature of Debye–Scherrer cones is lower which reduces the intrinsic broadening. Consequently it is possible to include a wider axial range of data than would be possible using a fixed receiving slit, thus also improving the statistical quality of the data at higher diffraction angles. This approach was developed with data from two test experiments using a Pilatus detector with 172 µm pixels reading out at 67 Hz. With the installation of the highly performing Eiger detector, the correction of the angular scale and filtering on intrinsic width are now being applied systematically. Using the geometry of the multi-analyser stage refined from a pattern of a standard sample such as NIST LaB_6_ 660c, using *Topas-7* (Coelho, 2018 ▸), including channel angular offsets, roll aberrations for the analyser crystals, centreline and tilt of the detector, *etc*. (Fitch & Dejoie, 2021 ▸), the appropriate angular corrections are calculated and intrinsic-width filtering applied immediately after the data are recorded, and transferred to the data file (Fig. 3 ▸).

**Figure 4:**
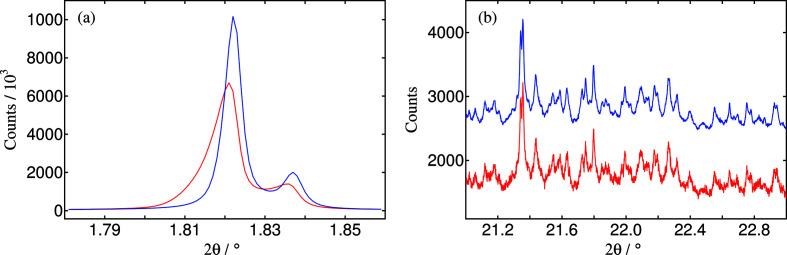
High-resolution powder-diffraction pattern of zeolite ZSM-5 at 35 keV showing (red) standard processing (Section 3.1.2) and summing of channels, *R*_exp_ = 0.0168; (blue) processing exploiting the axial resolution of the Eiger detector (filtering on an intrinsic width of 0.003°) to correct the 2θ scale, *R*_exp_ = 0.0096; illustrating (*a*) the reduction in low-angle asymmetry and improvement in angular resolution; (*b*) the improvement in the statistical quality of higher angle data by taking a greater axial contribution into the summation (blue curve offset by +1000 for clarity).

For each of the 13 channels, the counts in the (up to 512) regions of interest across the detector that comply with the intrinsic-width criterion are summed into bins of the same angular size as the actual data collection (0.0005° by default). Filtering with more than one intrinsic width, *e.g.* 0.002° and 0.003°, can be specified, producing for each a separate data file, allowing a choice to be made subsequently depending on the microstructural characteristics of the sample. The resulting data comprising the angle-corrected and width-filtered signals for each channel and each scan can then be added together to produce the equivalent step-scan data, using bins of an appropriate size as for the standard procedure described above, via a modified version of the summing program *id22sume*. The effects of the correction of axial divergence effects and increasing the axial acceptance with 2θ are illustrated in Fig. 4 ▸ for a typical sample, ZSM-5 zeolite.

### 3.2. Area-detector mode

There are two options for using an area detector to measure powder patterns more quickly than with high-resolution scanning, but with lower angular accuracy and resolution. This mode is useful for measuring data for PDF for glasses and for poorly crystalline materials where the microstructural broadening from the sample dominates the breadth of the diffraction peaks, neither of which benefit substantially from high angular resolution. It also allows data for PDF analysis to be measured more quickly while the sample is heated or cooled, *etc*., so following the evolution of local structure *in situ*.

#### 3.2.1. Perkin Elmer detector

When using the Perkin Elmer XRD1611 medical-imaging detector (41 cm × 41 cm, 16 Mpixels) to measure data for PDF analysis, it is positioned at a distance of ∼380 mm from the sample, which is as close as is physically possible with the sample mounted on the spinner on the axis of the powder diffractometer. The diffractometer has a second mounting point for the spinner on the ω circle, allowing a sample-to-detector distance of ∼140 mm, but this option is rarely, if ever, used. For PDF analysis, the detector is positioned so that the incident beam is directed towards its bottom corner, so that quadrants of the Debye–Scherrer rings are registered, and the maximum *Q* range is across the diagonal. For texture measurements, the detector can be positioned more centrally with respect to the beam so that full rings are measured. The detector is generally used at energies above 20 keV, and for PDF analysis an energy of 60–70 keV is appropriate to give *Q* > 25 Å^−1^. Because the detector has no energy discrimination, care must be taken if working at lower energies not to confuse signals from higher harmonics (λ/3, λ/4, *etc*.) with those from the primary wavelength.

Before being used, the detector is calibrated with regard to its distance from the sample, its tilts and point of normal incidence with respect to the incident beam, and possibly the wavelength. The geometric parameters are obtained by measuring the diffraction pattern of a standard sample such as NIST LaB_6_ using a wavelength already calibrated in high-resolution mode, and refining the distance and tilts, *etc*. with *pyFAI-calib* from the *pyFAI* suite (Kieffer *et al.*, 2020 ▸). These are then fixed and, if the wavelength is changed, its value is refined against a pattern from the standard sample measured at the new wavelength.

The detector is run in a continuous cycling mode with images read from it as required. This mode keeps the detector operating under a constant duty cycle, and helps in stabilizing its temperature and background noise to the image, which can vary if it is allowed to become dormant. Because the background can evolve with temperature and time, and because there is a certain afterglow, the detector is run in a mode whereby an image is recorded without beam, then with beam, and the dark background image is subtracted from the light image, which is recorded. A fast local X-ray shutter switches between these states. The shortest exposure time is 0.27 s, and, with electronic, background and processing overheads, the maximum frequency of recording patterns is ∼0.67 Hz, although the image-acquisition routine can increase the ratio of light to dark images to increase the overall rate. The maximum exposure time is 5 s and care must be taken not to saturate pixels, which have a depth of 16 bits (65535 counts), with strongly scattering samples. Normal operation involves taking a series of images from the sample, anything from 20 to 2000 frames depending on the nature of the sample and the study, and then summing them, using an in-house Python script (*sum2D_id22*) or *pyFAI-average*. The one-dimensional powder pattern (intensity versus 2θ or *Q*) is extracted from the summed images by integrating around the rings via *pyFAI-integrate*, taking into account the calibrated distance, point of normal incidence and tilt parameters.

#### 3.2.2. Stand-alone Eiger

The 13-crystal multi-analyser stage can be removed as a unit allowing the Eiger detector to be used as a standard 2D pixel detector, covering ∼25° at 680 mm from the sample. The detector is still attached to the detector arm so can be positioned at any angle within its nominal angular range. By taking overlapping images at several angles, measurements to high diffraction angles can be made more quickly than in the high-resolution scanning mode. This use of the Eiger detector is an alternative to the medical imaging detector for PDF studies, allowing data to be measured to high *Q* values without needing to change the wavelength to ≥60 keV. It takes longer to measure at several different 2θ angles than with the fixed Perkin Elmer detector; however, the intrinsic electronic background is lower. The choice is made depending on the nature of the sample and the aims of the study. The *pyFAI* suite is used for calibrating the detector’s spatial parameters and for integrating and combining images. If set at a fixed angle, images can be collected at a rate of up to 1 kHz in difference mode (2 kHz with a single energy threshold), allowing fast processes to be followed over ∼25° 2θ. Comparison of the 110 and 500/430 reflections of LaB_6_ measured in this mode *versus* high-resolution mode are shown in Fig. S1 of the supporting information.

### 3.3. Performance

#### 3.3.1. Resolution

**Figure 5:**
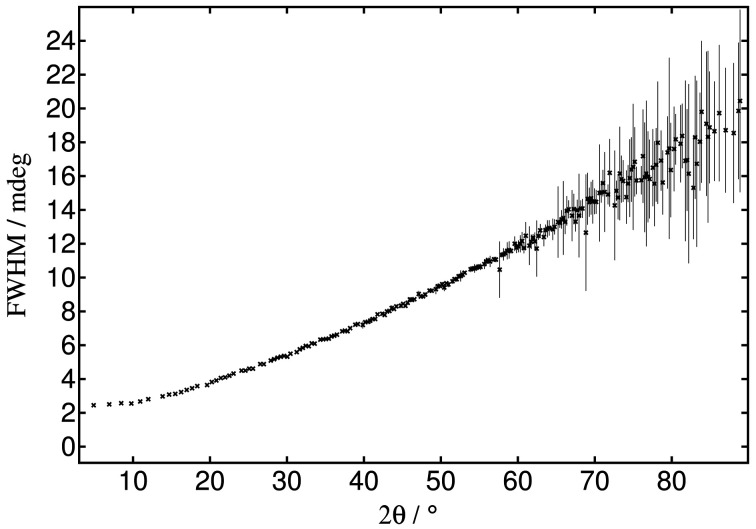
FWHM with error bars (σ) versus 2θ for peaks from NIST standard LaB_6_ 660c at 35 keV fitted individually with a symmetric *more_accurate_Voigt* function using *Topas*. The background was fixed at the values from the Rietveld refinement shown in Fig. 6 ▸.

For a high-resolution powder-diffraction beamline the theoretical peak widths can be calculated considering the divergence of the beam from the source and the Darwin width of the monochromator crystals which together define the bandpass (Δλ/λ) of the radiation incident on the sample, as well as the angular acceptance of the analyser crystals (Sabine, 1987*a* ▸,*b* ▸; Wroblewski, 1991 ▸; Masson *et al.*, 2003 ▸), also taking account of other optical elements in the beamline (Gozzo *et al.*, 2006 ▸). Pragmatically, the widths of the peaks from a standard sample of high crystallinity, such as NIST LaB_6_ 660c, can conveniently be used to measure the characteristics. Fig. 5 ▸ shows the results of fitting a symmetric Voigt function with *Topas* to each of the peaks from LaB_6_ measured at 35 keV following the correction for axial divergence and combination of the 13 channels, as described above, filtering on an intrinsic width of 0.002°. The peak widths (FWHM) vary from 0.0024° for the 100 reflection at 4.9° 2θ, corresponding to Δθ/θ of 5 × 10^−4^, to 0.02° around 90° 2θ, a Δθ/θ of 2 × 10^−4^.

**Figure 6:**
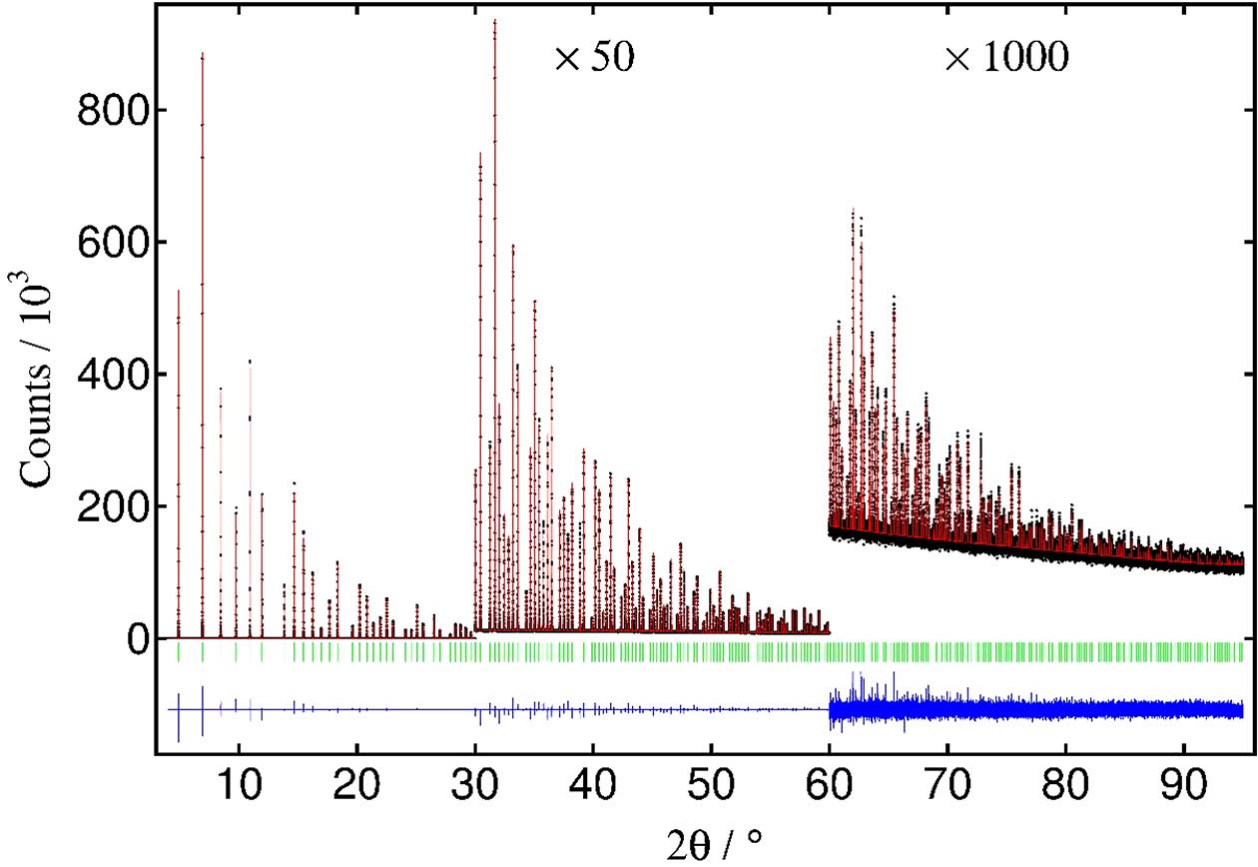
Rietveld refinement of NIST 660c LaB_6_ (35 keV) using *Topas*, *R*_wp_ = 0.0427, *R*_exp_ = 0.0293, χ^2^ = 1.46.

A Rietveld fit against the LaB_6_ 660c pattern is shown in Fig. 6 ▸. The calculated pattern was modelled with a Voigt peak-shape function (*more-accurate-Voigt*) with a *Simple_Axial_Model* for the slight intrinsic asymmetry resulting from the residual axial divergence due to the finite sizes of the incident beam and the receiving pixel (Fitch & Dejoie, 2021 ▸).

#### 3.3.2. PDF applications

We have compared the PDFs obtained from data collected with the 2D imaging detector and in high-resolution mode using increasing axial acceptance with 2θ. A mixture of three standard crystalline materials (CeO_2_, TiO_2_ and ZrO_2_ in equal parts by mass) was prepared. Total scattering measurements of the sample and the empty 0.5 mm-diameter borosilicate-glass capillary were performed with the Perkin Elmer detector, and in high-resolution mode applying corrections for axial divergence. For the former, the beam with an energy of 70 keV was focused on the detector at a distance of 380 mm from the sample. 1500 images (which corresponds to around 1 h data collection including dark frame and dead-time) were collected with an exposure time of 1 s each in 16-bunch mode (75 mA ring current). The 2D images were combined and converted to the powder-diffraction pattern using the *pyFAI* suite. For the high-resolution data, a nominally 1 mm × 1 mm beam with an energy of 35 keV was used scanning between −11° and 133° 2θ at 2.4° min^−1^ (60 min scan) in one of the ESRF ring’s 200 mA modes. The data from the 13 channels were combined, correcting the angular scale for axial divergence and filtering on an intrinsic peak width of 0.003°.

**Figure 7:**
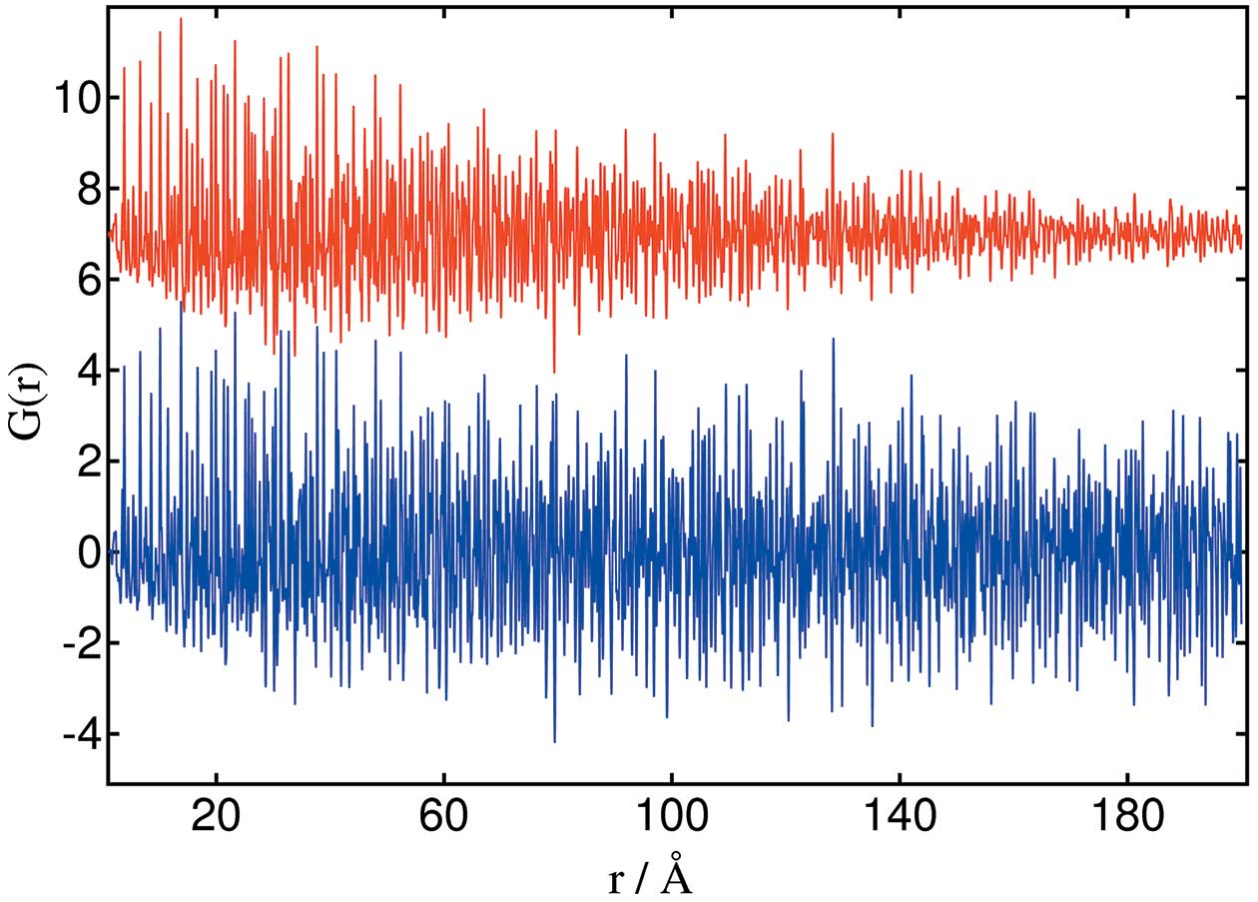
Pair distribution functions obtained from data collected with (blue) the high-resolution setup and (red, offset by +7) the Perkin Elmer detector. (See Fig. S2 of the supporting information for an expanded view for *r* up to 30 Å).

PDFs were calculated using the *PDFgetX3* software (Juhás *et al.*, 2013 ▸), imposing *q*_max_ and *q*_maxinst_ at 28 Å^−1^ and *rpoly* at 1.67. Fig. 7 ▸ shows the PDFs obtained using data collected with the two strategies. As is well known (Egami & Billinge, 2012 ▸), higher reciprocal space resolution reduces damping of the PDF signal at larger distances, allowing a better study of long-range atomic order. This illustrates a limitation of using a low-resolution 2D detector for PDF analysis, as in this particular case where the observed damping is not due to the sample.

In high-resolution mode, the increase in axial acceptance with diffraction angle leads to an improvement in statistical quality of the data at increasing angle as compared with a fixed-slit configuration, with benefit for studies requiring high-quality high-*Q* data such as PDF analysis. To assess how much time is required to obtain data for PDF analysis for crystalline and less-well crystalline samples, for which the high-*Q* scattering is intrinsically weaker, two samples with different particle sizes were chosen: the mixture of three crystalline materials (CeO_2_, TiO_2_ and ZrO_2_) mentioned above, and nano TiO_2_ (particle size < 100 nm, Sigma-Aldrich 637262). Data were collected at 35 keV in 200 mA mode. The samples were each scanned between −11° and 133° 2θ at 9.6 and 2.4° min^−1^, corresponding to collection times of 15 and 60 min, respectively, with an additional scan (30 min for the more-crystalline sample, and 120 min for the nano-crystalline titania). Empty capillaries were also measured. PDFs were obtained as above using *PDFgetX3*.

**Figure 8:**
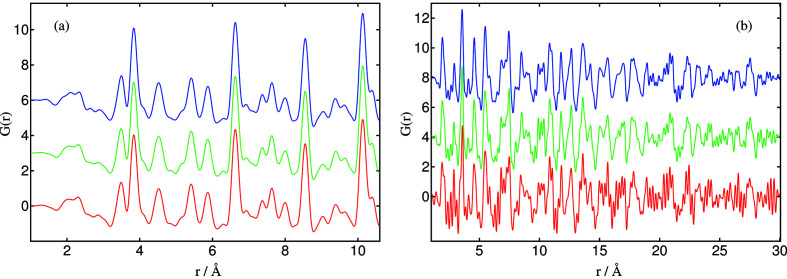
PDFs from high-resolution data measured for different times (*q*_max_ = 28 Å^−1^); (*a*) mixture of crystalline CeO_2_, TiO_2_ and ZrO_2_ (red, 15 min; green, 30 min; blue, 60 min); (*b*) nano-titania, size < 100 nm (red, 15 min; green, 60 min; blue, 120 min); no significant differences are observed comparing PDFs of the crystalline sample, even with a short acquisition time. This is evidently more relevant for the nano sample, where the PDF obtained in 15 min is strongly affected by the statistical noise at lower and higher *r*.

Fig. 8 ▸ shows the PDFs obtained with the different collection times. No significant differences in PDF are observable for the crystalline sample, implying that even 15 min could be enough to obtain a suitable PDF with the newly developed mode of data acquisition. In the case of nano TiO_2_, however, the broadening due to the reduced size of the particles and its effect on the higher-angle and diffuse scattering implies that a longer acquisition time is required (at least 60 min). For both samples, however, the results presented suggest that the newly developed mode of data acquisition, owing to the higher statistics at high *Q*, reduce significantly the time necessary to obtain a suitable PDF in the high-resolution setup as compared with using a fixed axial receiving slit.

#### 3.3.3. Main applications

Experiments predominantly involve the study of structure in materials (often while the sample is heated, or cooled, or exposed to different gases, *etc*.), especially the solution and/or refinement of crystal structures, or investigation of short-range correlations via pair distribution function analysis, which also includes poorly crystalline samples. High-resolution studies for crystal structure analysis are frequently carried out at 31 keV or 35 keV, unless there is reason to do otherwise, such as to avoid being too close to the absorption edge of an element such as Sn, Sb, Te, I, *etc*., or to employ anomalous scattering. PDF measurements are also possible with this setup, scanning to 2θ angles above 90° to have an adequate range in *Q*. The capability to increase the axial acceptance with angle up to the effective width of the detector improves the statistical quality of the high-angle data which can now usually be measured in a single scan, whereas before the installation of the Eiger detector it was necessary to scan these regions multiple times. Studies of substances with very large unit cells such as proteins (*e.g.* Karavassili *et al.*, 2017 ▸) are possible and are conducted at lower energies, 9.5 keV (1.3 Å wavelength), to allow the lowest-angle peaks to be seen above the 2θ range hidden by the beam stop (up to around 0.5°). The correction of asymmetry due to axial divergence and the consequent improvement of angular resolution that is possible with the Eiger detector are particularly important at low diffraction angles, though these improve the powder pattern over its whole range. The wide choice of wavelengths means that a *K* or *L* absorption edge is accessible for all elements beyond Cr, thus allowing anomalous scattering to be employed to enhance sensitivity to the arrangement of selected element(s) in a sample. High-resolution studies can be conducted in general up to around 60 keV. At higher energies, the Bragg angle onto the analyser crystals falls below 2°, and radiation can then pass between neighbouring crystals with adverse effects on the diffraction pattern’s background. Other studies that use high-resolution powder diffraction include quantitative and microstructural analyses. High-resolution data leads to a clearer distinction between of the components of mixtures of many crystalline phases, some maybe present in low concentration. The low instrumental contribution to the diffraction peak shapes means that sample effects dominate, allowing the separation of characteristics such as crystallite domain size and micro-strain from the analysis which is aided by being able to measure a large number of diffraction peaks to high diffraction angles (Scardi *et al.*, 2018 ▸).

## 4. Conclusion

ID22 is a versatile high-resolution powder-diffraction beamline working in the energy range 6–75 keV. It operates predominantly in high-resolution mode, benefitting from the highly collimated and monochromatic beam incident on the sample coupled with a 13-channel multi-analyser stage and Eiger detector. Lower-resolution modes are also available, via a Perkin Elmer medical-imaging detector or the Eiger detector without the multi-analyser stage, allowing faster data acquisition at the expense of angular resolution and accuracy. The beamline has a range of dedicated sample environments integrated into the beamline’s control system, allowing capillary specimens to be studied over the temperature range of 4 K to 1600°C, and under pressures of 0–100 bar of non-corrosive gas. The open access to the diffractometer allows complex sample environments to be accommodated for setups brought by visiting user groups.

The Perkin Elmer medical-imaging detector is used predominantly for PDF analysis at energies of 60 keV or higher. With a fixed detector in which the whole diffraction pattern is registered at the same time, data acquisition is faster than in the high-resolution scanning mode, but the angular resolution and accuracy are inferior. Nevertheless the majority of PDF experiments have been conducted using this approach, especially if the evolution of the sample is being followed *in situ*. Complementary use of both high-resolution scanning and of the 2D detector is also possible in the same experiment for high-quality data for crystallographic analysis coupled with investigation of short-range order by PDF analysis, possibly coupled with measurements using other beamlines or measurement techniques to probe more deeply into a material’s structure and properties (*e.g.* Scavini *et al.*, 2022 ▸).

## Supplementary Material

Sections S1 and S2, including Figures S1 and S2. DOI: 10.1107/S1600577523004915/vy5010sup1.pdf
