# Supplementary material for: ID22 – the high-resolution powder-diffraction beamline at ESRF
Source: J Synchrotron Radiat. 2023 Jul 18;30(Pt 5):1003–12. doi: 10.1107/S1600577523004915 (PMC10481261; doi:10.1107/S1600577523004915)
Supplement: Supplementary file 1 [file s-30-01003-sup1.pdf]

JOURNAL OF SYNCHROTRON RADIATION

Volume 30 (2023)

Supporting information for article:

ID22, the high-resolution powder-diffraction beamline at ESRF

Andrew Fitch, Catherine Dejoie, Ezio Covacci, Giorgia Confalonieri, Ola Grendal, Laurent Claustre, Perceval Guillou, Jérôme Kieffer, Wout de Nolf, Sébastien Petitdemange, Marie Ruat and Yves Watier

S1. Update to Fitch and Dejoie (2021)

In Fitch and Dejoie (2021) a method was developed for correcting the angles given by the nominal position of the diffractometer arm, to the true 2θ position defined by diffraction from the sample, via the axial position of the photon arriving on the detector. As part of that procedure equations (17) and (22) of that paper were derived which take the form of

$$X\cos(A) + Y\sin(A) = Z \tag{S1}$$

where X, Y and Z are combinations of trigonometric functions of various angular quantities and A is related to the angle of the diffractometer arm, $(2\Theta - \theta_a)$, or to the true diffraction angle 2θ at the sample, quantities that are being sought in the procedures; (2Θ is the mechanical angle of the detector arm and θ_a is the Bragg angle of the analyser crystal). The equations were manipulated to yield quadratic equations, e.g. equation (19), with solutions, equations (20) and (22), in the form,

$$\cos(A) = \frac{2ZX \pm \left[4Z^2X^2 - 4(X^2+Y^2)(Z^2-Y^2)\right]^{1/2}}{2(X^2+Y^2)} \tag{S2}$$

The desired quantity A was obtained by taking the inverse cosine. However a computational problem arises if $(2\Theta - \theta_a)$ is negative (i.e. at low-angle regions of the powder diffraction pattern) because $\cos(A) = \cos(-A)$ and the positive rather than the negative solution is returned. A method for dealing with this was given in the supplementary information, section S4, involving numerically calculating a second derivative. We now have a more convenient approach avoiding this issue. Equation (S1) can be solved to yield (thanks to Wolfram Alpha)

$$A = 2\tan^{-1}\left(\frac{Y \pm \left[X^2+Y^2-Z^2\right]^{1/2}}{X+Z}\right) \tag{S3}$$

$\mathrm{Tan}^{-1}(x)$ is a one-to-one function with values in the range –π/2 – π/2, thus (S3) covers a continuous range of angles of –180 – 180°. The relevant function in the Topas refinement becomes

```
fn dd(om, fx, fy) {
def xx = Sin(fx) Sin(fy) Cos(2 Th) - Cos(fx) Sin(2 Th) Cos(om) ;
def yy = Cos(fx) Cos(2 Th) + Sin(fx) Sin(fy) Sin(2 Th) Cos(om) ;
def zz = Sin(fx) Cos(fy) Sin(om) Sin(2 Th) - Sin(alpha) ;

def TT = 2 ArcTan((yy - Sqrt(xx^2 + yy^2 - zz^2)) / (xx + zz))  ;
def Delta = (TT + alpha - 2 Th ) ;   ' Delta is in radians
return Delta ; }
```

S2. Comparison between data collected with and without analyser crystals.

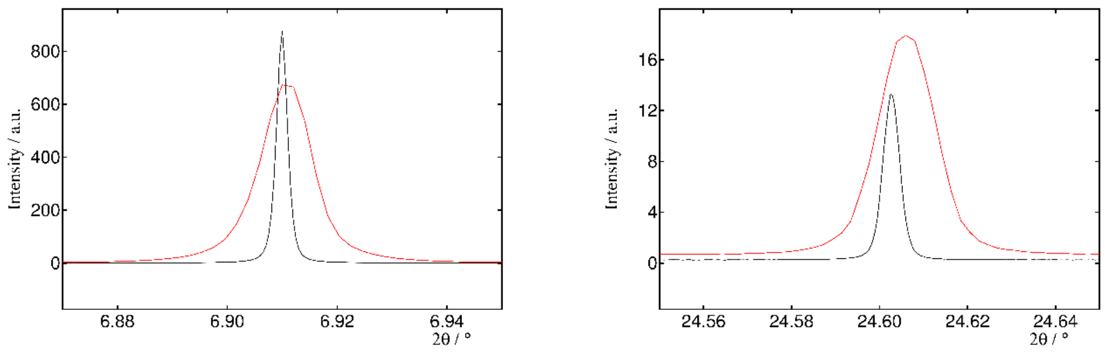

Figure S1 Comparison of the (*left*) 110 and (*right*) 500/430 peaks of NIST 660c LaB_6 measured in a 0.5 mm diameter capillary at 35 keV; (*black*) with the multi-analyser stage, λ = 0.354243 Å, FWHM of 0.0025° and 0.0045°, respectively; (*red*) without the multi-analyser stage, beam focussed on the Eiger detector at 676.4 mm from the sample, see main section 3.2.2, λ = 0.354294 Å, FWHM of 0.0115° and 0.0148°, respectively.

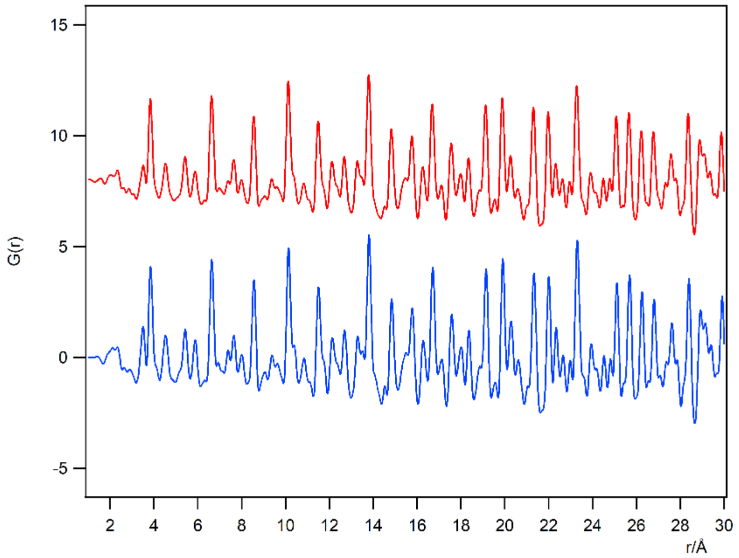

Figure S2 Pair distribution functions obtained from data collected with (*blue*) the high-resolution setup; and (*red*, offset by +7) the Perkin Elmer detector; expanded low-r part of main Fig.7. Evidently the two PDFs are very similar.

References

Fitch, A. & Dejoie, C. (2021). *J. Appl. Cryst.* **54**, 1088–1099.
